# The Brainfit study: efficacy of cognitive training and exergaming in pediatric cancer survivors – a randomized controlled trial

**DOI:** 10.1186/s12885-017-3933-x

**Published:** 2018-01-03

**Authors:** Valentin Benzing, Noëmi Eggenberger, Janine Spitzhüttl, Valerie Siegwart, Manuela Pastore-Wapp, Claus Kiefer, Nedelina Slavova, Michael Grotzer, Theda Heinks, Mirko Schmidt, Achim Conzelmann, Maja Steinlin, Regula Everts, Kurt Leibundgut

**Affiliations:** 1 Institute of Sport Science, University of Bern, Bern, Switzerland; 2 Division of Neuropaediatrics, Development and Rehabilitation, University Children’s Hospital Bern, Inselspital, Bern University Hospital, University of Bern, Bern, Switzerland; 3 Insitute of Psychology, University of Bern, Bern, Switzerland; 4 Division of Diagnostic and Interventional Neuroradiology, Inselspital, Bern University Hospital, University of Bern, Bern, Switzerland; 5 Division of Pediatric Oncology, University Children’s Hospital Zurich, Zurich, Switzerland; 6 Center for Cognition, Learning and Memory, CCLM, University of Bern, Bern, Switzerland; 7 Division of Pediatric Hematology and Oncology, University Children’s Hospital Bern, Inselspital, Bern University Hospital, University of Bern, Bern, Switzerland

**Keywords:** Childhood cancer survivors, Brain tumor, Working memory training, Physical exercise, Physical training, Active video gaming

## Abstract

**Background:**

Cancer survival comes at a price: pediatric cancer survivors bear a high risk for a wide range of cognitive difficulties. Therefore, interventions targeting these difficulties are required. The aim of the present clinical trial is to extend empirical evidence about efficacy of cognitive and physical training in pediatric cancer survivors. It is hypothesized that early cognitive and physical interventions affect the remediation of pediatric cancer survivors in terms of improved executive functions (primary outcome). Additional positive effects of cognitive and physical intervention to other areas such as memory and attention are expected (secondary outcome). Changes in cognitive performance are expected to be associated with structural and functional changes in the brain.

**Methods:**

Overall, 150 pediatric cancer survivors and 50 matched controls will be included in this trial. The cancer survivors will be randomly assigned to either a computerized cognitive training, a physical training (exergaming) or a waiting control group. They will be assessed with neuropsychological tests, tests of sport motor performance and physical fitness before and after 8 weeks of training and again at a 3-months follow-up. Moreover, neuroimaging will be performed at each of the three time points to investigate the training impact on brain structure and function.

**Discussion:**

With increasing cancer survival rates, evidence-based interventions are of particular importance. New insights into training-related plasticity in the developing brain will further help to develop tailored rehabilitation programs for pediatric cancer survivors.

**Trial registration:**

KEK BE 196/15; KEK ZH 2015–0397; ICTRP NCT02749877; date of registration: 30.11.2016; date of first participant enrolment: .18.01.2017.

## Background

Cancer is the leading cause of death by disease in children and adolescents aged 5–15 years [1]. Advances in early diagnosis and improved treatment approaches have led to an increase in long-term survival rates of up to 80% [2]. However, survival of pediatric cancer comes at a price: An increasing amount of literature indicates that the most frequent pediatric cancer diagnoses are associated with late effects including neurocognitive deficits and intellectual decline (e.g., [3–5]).

Cognitive functioning in pediatric cancer is affected by complex interactions between several factors such as age at onset or treatment modality (for review see [6]). For example, younger age at diagnosis and at treatment of central nervous system (CNS) tumor is associated with greater cognitive problems indicating that sequelae of cancer and treatment depends on the developmental status of the child [7]. The most common treatment for pediatric cancer include surgery, chemotherapy, and radiation therapy. All of these treatments do not solely target malignant cancer cells, but entail harmful effects to multiple organ systems, including the CNS [4]. Treatment related side effects such as neurotoxicity [8, 9] seem to be particularly harmful to specific cognitive processes such as the executive functions (EF) [10–12].

EF are of particular importance for academic achievement with executive dysfunction having far reaching consequences on survivors’ scholastic career and overall quality of life [13, 14]. There are three core EF (inhibition, shifting and working memory) that build the basis for higher order cognitive functioning such as planning or problem solving [15, 16]. Impairments in EF at an early stage after diagnosis will put the patients at risk to academically fall behind peers [3, 17, 18]. Thus, research recently focuses on developing adequate intervention and rehabilitation programs with the aim to alleviate cognitive impairments, facilitate the return to school and improve the long-term quality of life of pediatric cancer survivors (e.g., [4]). Although the neurocognitive impairments in pediatric cancer survivors imply the necessity to intervene as early as possible [6, 18], until now only very few intervention studies in this population are available.

Cognitive training programs have been used to address core cognitive deficits in a variety of individuals with developmental difficulties. Home-based, computerized cognitive training carries a minor burden because it can be completed flexibly at any time at home without adverse side effects [19]. Computerized cognitive training (e.g., Cogmed RM® [20]) is often based on a core cognitive function such as i.e. working memory and follows the assumptions that through repeated and intensive practice cognitive capacity can be increased. Several studies investigated the efficacy of such a working memory training in children and adolescents with atypical development such as attention deficit hyperactivity disorder (e.g., [20, 21]), traumatic brain injury, stroke [22, 23] or very preterm-born children [24]. There is supporting evidence for improvements on tasks that resemble the training task (near-transfer effects) following working memory training. However, transfer effects to other untrained cognitive domains (far transfer effects) are currently subject to a controversial debate (for more information see [25–28]).

Studies investigating working memory training in pediatric cancer survivors revealed promising results (for review see [5]). Hardy and colleagues showed significant improvement in visual working memory and in parent-rated learning problems in a pilot study with 20 pediatric cancer survivors after working memory training when compared to an active control group undergoing a non-adaptive intervention [29]. Working memory trainings, such as Cogmed RM®, were found to be feasible and a viable option to address cognitive late effects among pediatric cancer survivors [19, 30]. Although there are first encouraging results regarding working memory training, yet data are too limited to form “best practice” guidelines [5].

Physical exercise seems to be another promising approach to foster cognitive performance. Many studies indicate that physical exercise can have positive effects on a range of cognitive functions in typically developing children and adolescents [31–33]. Regularly performed physical exercise can alter brain functions responsible for cognition and behavior [31, 34, 35]. In particular, EF seem to benefit from physical training (e.g., [36–38]). Recently, there has been an increasing interest in qualitative factors of physical exercise such as cognitive engagement (e.g., [39–41]), because they likely influence cognition in a positive way [32, 39, 42]. A recent study was able to demonstrate that a 6-week cognitively demanding sports game intervention for school children, but not a pure endurance training, yielded significant intervention effects on shifting, a core dimension of EF [43]. The underlying assumption is that cognitively engaging physical activities also train brain regions that are used to control higher order cognition [34, 42, 44]. Hence, physical exercise should ideally not only challenge the body but also the mind.

An innovative combination of a physically and cognitively demanding training at home can be achieved with exergaming [45]. Exergaming is a portmanteau of “exercise” and “gaming” [46], which enables individuals to physically interact with a virtual environment. In a gamified fashion, the individual has for example to avoid obstacles without touching them by jumping from left to right. The quantitative (intensity, duration = e.g., faster obstacles) and qualitative physical exercise characteristics (modality = e.g., jumping, running) can be modulated, allowing to go “beyond simply moving to moving with thought” [47]. Up to date, there are few studies investigating the relationship between exergames and cognition [37, 41, 48–51]. In pediatric cancer survivors, first positive results of physical exercise on quality of life, body composition and physical activity have been described [52, 53]. However, high quality studies including larger samples are needed [52, 54]. Although exergaming enables a training under highly controlled conditions at home, to our knowledge, no study to date examined the impact of physical exercise or exergaming on cognitive performance in pediatric cancer survivors.

The literature suggests that training related changes in brain structure and function can occur with cognitive and physical training [35, 55]. Although there seems to be evidence that they might facilitate a positive effect on cognition, the underlying mechanisms remain unclear. Brain imaging therefore might be a valuable method to identify neuronal effects following working memory training or physical exercise. In adults, studies on working memory training and physical exercise revealed brain changes in structure and function (for reviews see [35, 55]). There is, evidence for increases of brain activation (stronger neural response and greater task related activity), decreases of brain activation (increased neuronal efficiency) or a combination of both [55]. Furthermore, there seems to be an increased functional connectivity for example in the default mode network and in the executive network following physical exercise [56]. Studies on both, working memory training and physical exercise, detected an increase in cerebral blood flow following training [35, 55]. Besides functional changes, structural changes in gray or white matter have been found. However, there seems to be no clear single pattern of results regarding the neural effects of cognitive and physical training, pointing towards highly dynamic plastic processes underlying cognitive change [55].

In pediatric cancer survivors, only very few studies on working memory training or physical exercise are available. A study on working memory training suggests alterations in the functional network of working memory [30] while physical exercise has an impact on white matter and hippocampal volume [57]. However, the relationship between cognitive or physical training and neuroplasticity in pediatric cancer is far from understood. Therefore, more studies are needed to investigate the neural mechanisms of training.

### Study aims and hypotheses

The purpose of this study is twofold: First, the availability of well-designed, child-friendly, and evidence-based training is of major clinical relevance and will contribute to the prevention of a further decline of cognitive functions and scholastic problems. Therefore, this study will compare the efficacy of two different trainings aiming to foster cognitive performance in pediatric cancer survivors. As primary outcome, we hypothesize that both trainings (computerized working memory training and exergaming) lead to improvements in core EF performance (inhibition, shifting and working memory) compared to a control condition. As secondary outcome, near and far transfer effects of both trainings are expected immediately after the training and at 3-months follow-up.

Second, the detection of training-induced changes in brain structure and function will give insight into the training related plasticity of the child’s brain. As further secondary outcome, the relationship between training related cognitive change and training related change in brain structure and function will be examined.

## Methods

### Design

**Fig. 1:**
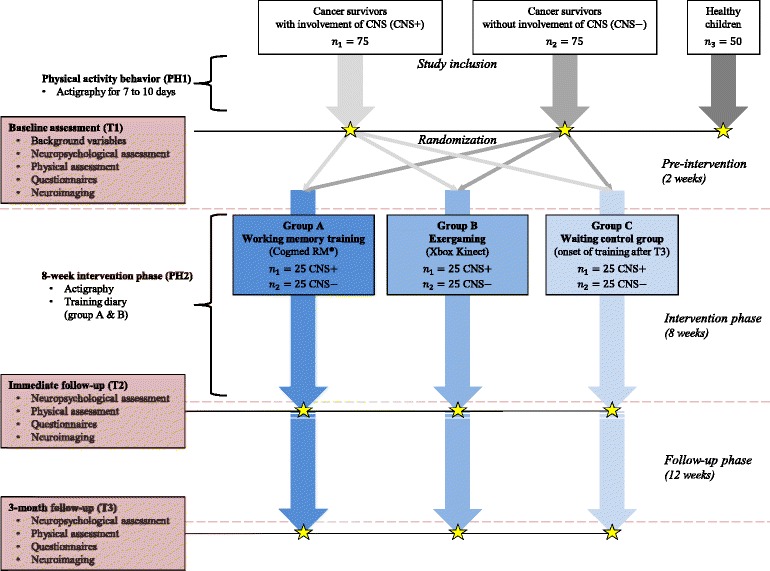
Detailed study design for the two intervention groups (Groups A and B) and the waiting control group (Group C)

The study is designed as a randomized stratified controlled trial including three experimental groups (computerized working memory training, exergaming, waiting control group) consisting of pediatric cancer survivors and a healthy control group without intervention (matched for age, gender and socioeconomic status). For cross-sectional evaluation of cognitive performance, cancer survivors and healthy participants will be compared in a baseline assessment. For longitudinal evaluation, only cancer survivors will be randomly assigned to either intervention group A (computerized working memory training), intervention group B (exergaming) or the waiting control group C (see Fig. 1). Cognitive and physical assessment will be carried out before the interventions and the waiting period (baseline assessment; T1) and will be re-performed with all participants after 8-weeks at immediate follow-up (T2) and at a 3-month follow-up (T3). Structural and functional imaging will be performed at each time-point (T1-T3).

### Participants

#### Cancer survivors

In total, 150 children and adolescents aged 7–16 years with a previous diagnosis of cancer with or without CNS involvement (CNS+ and CNS-) in the past ten years and terminated their treatment (surgery, radiation, and/or chemotherapy) at least 12 months prior to participation will be included in the study. Cancer survivors will be recruited at two specialized pediatric university hospitals in Zurich and in Bern, Switzerland. To reduce the heterogeneity of the sample, participants with a history of cancer without CNS involvement and only surgical removal of the tumor without subsequent radiation and/or chemotherapy will be excluded from the study. The sample size was calculated for the primary outcome measures of inhibition, shifting (Color-Word Interference Test; retest-reliability = .77) and working memory performance (Block Recall Test; retest-reliability = .62), both will be assessed before and after the trainings and before and after the waiting period. Sample size calculation was performed using G*Power 3 [58], based on a repeated measures ANOVA with small effect sizes (six groups; two time points; statistical power = 80%; *α* = 0.05), resulting in a minimal sample size of 14 (for inhibition, shifting) and 22 (for working memory) in each group. In order to compensate for losses/drop outs, sample size was defined at 25 patients per group.

#### Healthy controls

50 healthy children and adolescents (matched for age, gender and socioeconomic status) will be included in the baseline assessment and will serve as a healthy control sample without intervention. They will be recruited mainly via siblings of cancer survivors and through flyers in the hospital and its neighborhood.

### Interventions

All 150 participants with a history of cancer will be randomly assigned to one of three experimental conditions (A, B, and C). The participants in the experimental conditions (A, B) will absolve three training sessions per week for a period of 8 weeks of their respective training; each session takes about 45 min. The difficulty level of the trainings (A, B) is adaptive and adjusted based on the user’s performance. Each participant will thus absolve a total of 25 training sessions (including the supervised first training session) of individually adapted difficulty levels. The training will be supervised by training aides (parent or guardian) and trained coaches. In both trainings, a coach will provide weekly supervision via phone call to the child’s home.

***Group A*** *(n*_*1*_ *= 25 CNS+, n*_*2*_ *= 25 CNS-*) will undergo a computerized working memory training program (Training A), that targets the storage as well as the processing of verbal and visual-spatial components taxing working memory capacity (e.g., corsi block tasks, rotating exercises). The participants will receive individual working memory training (Cogmed RM® [20]), which allows participants to train at home and allows the coach to review and monitor the results of the training online.

***Group B*** *(n*_*1*_ *= 25 CNS+, n*_*2*_ *= 25 CNS-)* will receive a physical exercise training (exergaming). The physical training will be performed at home using the XBOX Kinect (Microsoft, Redmond, WA). The used device is able to project the player on the TV screen by means of a camera which enables the player to engage in different virtual realities. The physical activity level can be playfully increased and individually adapted. The exergame (Shape UP, Ubisoft, Montreal) will comprise activities such as jump’n’run activities, coordinative exercises, and dance-like activities which has been shown to be cognitively and physically challenging [41]. All materials (XBOX Kinect, game, if necessary a TV) will be provided to the participants.

***Group C*** *(n*_*1*_ *= 25 CNS+, n*_*2*_ *= 25 CNS-)* will serve as a waiting control group and will receive either the physical or the computerized working memory training program after completion of the immediate follow-up assessment.

### Outcome measures and time-points of assessments

The study is divided into five sections (PH1 & PH2; T1–T3; see in Fig. 1). Participants with a history of cancer will participate in each section, whereas healthy control participants will only participate in PH1 & T1.

Physical activity behavior (PH1): In order to monitor the participants’ physical activity behavior before the baseline assessment, all participants will receive an accelerometer (Actigraph GT9X). The device will be sent to the participants 7–10 days prior to the baseline assessment.

#### Baseline assessment (T1)

The baseline assessment will take place at the Children’s University Hospital, Inselspital in Bern approximately seven days after handling over the accelerometer. Background variables including height, weight, socioeconomic status (income, highest parental education, family affluence scale [59]), and physical activity behavior (BSA [60]) will be obtained. A standardized neuropsychological assessment, a physical assessment, questionnaires and neuroimaging will be performed at the baseline assessment (see Fig. 1).

#### Neuropsychological assessment

The following cognitive functions will be assessed at all three time points (T1-T3): executive functions (Color-Word Interference Test of the Delis–Kaplan Executive Function System™ (D–KEFS™) [61]; visuospatial working memory (Block Recall Test of the Working Memory Test Battery for Children (WMTB-C) [62]; verbal working memory (Number Recall, Word Order, Atlantis, Rover of the German version [63] of the Kaufman Assessment Battery for Children, Second Edition) [64]; processing speed (Coding, Cancellation, Symbol search tests of the German Version of the Wechsler Intelligence Scale for Children, Fourth Edition) [65]. Only at the baseline assessment, IQ and manual dexterity will be assessed as control variables (Test of Nonverbal Intelligence, fourth edition (TONI-4) [66], Grooved Pegboard test [67]).

#### Physical assessment

Sport motor performance will be measured using the German motor performance test [68]. In addition, the fitness status of the participants will be administered using a VO2_max_ test (Godfrey protocol) [69].

#### Questionnaires

Several psychological domains will be assessed by means of questionnaires (German versions), which will be filled out by the participants and their parents: Quality of life (inventory of quality of life for children and adolescents [70], the kidscreen – Health Related Quality of Life Questionnaire [71]), psychological attributes such as emotional symptoms, conduct problems and others (Strengths and Difficulties Questionnaire (SDQ) [72]), physical self-description (short version of the Physical Self-Description Questionnaire (PSDQ-S) [73]), the questionnaire on resources in children and adolescents (FRKJ 8–16 [74]) and everyday executive functions (inhibit, shift, emotional control and working memory scales of the Behavior Rating Inventory of Executive Function (BRIEF) [75]).

#### Neuroimaging

Magnetic resonance imaging (MRI) of the brain will be performed with all participants fulfilling the required safety standards for scanning (e.g. no dental braces, magnetic stimulators, pumps or heart pacemaker). All participants will undergo MRI without anesthesia or contrast agents. Neuroimaging will be performed at the Institute of Diagnostic and Interventional Neuroradiology, University Hospital of Bern, Inselspital. The staff has extensive experience with pediatric participants from earlier neuroimaging studies [76–78].

#### Structural imaging

All MRI images will be acquired using a 3 Tesla Siemens Magnetom Prisma, VE11C Scanner (Siemens Erlangen, Germany), equipped with a 64-channel head coil. Anatomical imaging will be performed using a 3-D T1 magnetization prepared rapid gradient echo (MPRAGE) sequence for acquisition of T1-weighted structural brain imaging (acquisition time TA: 4:33 min, repetition time TR = 1950 ms, echo time TE = 2.19 ms, slices per slap 176, field of view FoV 256, 1 mm voxel resolution).

#### Functional imaging

For the investigation of resting state functional connectivity, a multi-band EPI sequence from the University of Minnesota (Center for Magnetic Resonance Research), TA: 5:06 min, distance factor 0% (gap 0 mm), excitation pulse duration 5120 us, flip angle 30° (avoiding rf-clipping; is in the order of the Ernst angle for TR = 300 ms and T1 of grey matter) will be used. Functional magnetic resonance imaging of working memory will be administered using an established paradigm assessing the visuospatial working memory network [78, 79]. For the examination of fractional anisotropy (structural connectivity), a diffusion sequence (MDDW) with 12 directions, slice and PE acceleration 2 and 2 resp., voxel size 2.2 mm iso, slices 54, TA: 1:37 min will be used. For the investigation of the arterial blood flow, an QII FAIR 3D–ASL (arterial spin labeling) will be administered (TA: 4:59 min PM: REF Voxel size: 1.5 × 1.5 × 3.0 mm, slices per slab 40, TR = 4600 ms, TE = 16.18 ms, post-labeling (inversion time) varies depending on patient and age, bolus duration 700 ms, inversion time 1500-2000 ms). For quantification purpose of arterial blood flow, a M0 run is added. Total scanning time will be 25 min. To minimize head motion, a head support system consisting of two pillows positioned on each side of the head will be used. Earplugs will reduce the scanner noise. All MR scans will be subjected to a radiological evaluation by an experienced neuroradiologist.

8-week intervention phase (PH2): During the intervention phase, the participants in the two training groups will train three times a week for 45 min (for further information see intervention section). During a supervised first training session, enjoyment, affect, arousal [80], cognitive and physical exertion [81] and the heart rate will be measured. The frequency of training will be recorded using a training diary. In addition, the physical activity will be recorded by an accelerometer (over a period of 8 weeks).

Immediate follow-up (T2): Pediatric cancer survivors will be re-assessed with the same tests and MRI protocols as in T1, shortly after termination of the 8-week intervention phase. If available, parallel test versions will be used for the assessment at this time-point and at follow-up. In addition to evaluate the overall acceptability and feasibility of the conditions, parents and participants will be given satisfaction questionnaires based on the questionnaires developed by Cox et al. [19].

3-month follow-up (T3): The three experimental groups will be re-assessed again at a 3-months follow-up with the same tests and same MRI protocol as in T1 and T2, to investigate the time dependent effect of the different training regimens.

### Randomisation and blinding

Stratification will be applied by etiology (CNS- or CNS+), age in years (7–9; 10–12; 13–16), center (Zurich or Bern) and nonverbal IQ (IQ < 93.5; IQ 93.5–106.5; IQ > 106.5) using the minimization randomization method [82] comprised in SecuTrial®. The allocation, enrolment and assignment will be carried out by RE. Participants with physical restraints (e.g. remaining paresis after surgery) who are unable to perform physical activities will be assigned to group A or C. Because this assignment breaks the randomization, these participants will be excluded from between-groups comparisons and will be analyzed only exploratively. Investigators assessing participants at T2 and T3 will be blinded with regard to treatment assignment of the participants. If there is any premature unblinding (e.g., accidental or due to a serious adverse event) the investigator has to promptly document and explain to the sponsor.

### Data analysis

The data collected will be tested as to their statistical properties and analyzed accordingly. The level of significance is set to *α* = 0.05. Normally distributed background and control variables will be compared between the groups (experimental vs. control group) using two-sample *t*-tests, and variables without normal distribution will be tested using Wilcoxon rank-sum test. Chi-squared test will be used for categorical variables. The diary entries and the execution data obtained from the XBOX/ Cogmed RM® during training sessions will be used to provide information about the focus and the frequency of the training. These scores will be presented as descriptive summaries. The frequency and duration of exercises during the intervention period will then be compared between the experimental groups.

#### Behavioral data

Raw scores of cognitive tests will be transformed to standard scores, *T*-scores or Intelligence quotient (IQ)-scores using age norms from the respective test manuals. Subsequently, to make results comparable among different tests, *z*-scores will be computed. In order to compare the baseline performance between patients and controls, Analyses of Variance (ANOVAs) will be calculated. To examine training effects, primary and secondary outcome variables of T1, T2 and T3 will be analyzed using Analysis of Covariance (ANCOVA) comparing the three groups, in order to increase statistical power and reduce possible bias [83]. To test whether inter-individual differences predict training or transfer effects, linear regression analyses will be performed (predictor variables i.e. gender, age, kind of cancer, cancer treatment methods, IQ).

#### Neuroimaging data

Pre- and post-processing of structural and functional data will be performed using SPM12 (Welcome Departement of Imaging Neuroscience, London) and MATLAB programs (MATLAB version 8.3). In order to perform functional connectivity analyses, the GIFT Toolbox will be used and within the framework of Independent Component Analysis (ICA) using the Group ICA Toolbox (GIFT software) [84] in order to compute the feature of the resting state network and the functional connectivity network. Functional magnetic resonance imaging of working memory will be analysed using SPM. After slice timing, spatially realignment and unwarping, data will be normalized using custom-generated pediatric reference data (TOM-toolbox; [85]). Images will be smoothed by a Full Width at Half Maximum Gaussian kernel. First level analyses will be conducted using the General Linear Model contrasting the active and baseline conditions. Resulting contrast images will be entered into a random-effect second level analysis, applying one-sample *t*-tests. The fractional anisotropy maps resulting from the diffusion sequence are provided by the Siemens software and can be used for further statistical evaluation of relative longitudinal comparisons on a single- subject level. ASL will be analyzed on a single-subject level; afterwards the resulting relative cerebral blood flow (rCBF) values will be used for further statistical group analyses.

#### Missing data

Missing data will be imputed for the respective analyses. Data missing completely at random will be replaced by Expectation-Maximization (EM) algorithm. If data is not missing completely at random, a multiple imputation will be applied.

#### Data management

Data will be stored and analyzed using Redcap, which is supervised by the Clinical Trials Unit of the Faculty of Medicine of the University of Bern and the Inselspital, Bern University Hospital., Switzerland. Redcap is a secure and reliable web application for building and managing databases. All electronic data will be stored in Redcap; all other data will be recorded in paper case report forms (stored in locked file cabinets) and subsequently electronically recorded.

#### Data monitoring

Data will be monitored regularly. Interim data analyses will be performed by the PI or his designees in order to regularly verify the quality of the data (e.g., exclude data loss or measurement errors due to technical problems). In case of adverse events, the following information will be collected: time of onset, duration, resolution, actions taken, assessment of the severity and of the relationship with the study intervention. No audits and inspections are planned in advance, however direct access to source documents will be permitted for purposes of audits and inspections to the Swiss ethics committee. The study documentation and the data will be accessible to independent auditors/inspectors and questions will be answered during inspections. All involved parties must keep the participant data strictly confidential.

## Discussion

Survivors of pediatric cancer frequently experience cancer-related cognitive sequelae [3, 4]. Despite steadily improved medical approaches, pediatric cancer is often followed by lifelong cognitive constraints, leading to significant academic and professional limitations and thus a reduced quality of life [4]. Scientific evidence for the potential negative cognitive impact of chemotherapy is emerging and seem alarming [12, 86]).

Cancer treatment has long known to be associated with harmful effects to multiple organ systems, including the CNS. The cognitive impairments caused by the therapeutic interventions (surgery, chemotherapy and/or radiation) may be caused by therapeutic interventions, because cancer treatment might damage healthy cells. They have been associated with a vulnerability of higher-order cognitive processes such as EF and in particular attention and working memory (e.g., [4, 6, 9, 17]). The question of interest therefore is whether early interventions can ameliorate the extent to which these late effects impair cognitive functioning of pediatric cancer survivors [6].

As discussed in the introductory section, some studies support the efficacy of computer based working memory training programs for children with attention and working memory deficits (e.g., [20, 21]. Studies on physical exercise also seem to yield cognitive improvements in children and adolescents (e.g., [31–33]). However, until now only very few intervention studies with pediatric cancer survivors have been published. Therefore, the investigation of different training methods in a group of children in need of support is of major importance. When administered early after treatment, such training programs might have the power to ameliorate or even prevent EF problems and thus academic failure upon return to school.

There are several advantages emerging from computerized interventions. Both trainings used in the present study (Cogmed RM® and exergaming) are comparably easy to implement and enable highly controlled conditions. In addition, they offer a direct form of reward, which occurs during and immediately after training via the feedback from the computer and the number of points scored. The trainings are presented in a child-appropriate form, which might be a promising approach to foster EF performance. Moreover, an advantage of computerized interventions is the adaptivity. The level is adjusted continuously, in order to create an optimal challenging training and avoid mental underload. Besides promising first results, our study seems to be the first to examine the effects of a physical exercise on cognitive functions in pediatric cancer survivors. In addition, it is the first study comparing cognitive and physical interventions in a population of pediatric cancer survivors.

A few limitations need to be mentioned, which each and altogether might affect study results. First of all, the recruitment of the study participants will take place in two specialized pediatric units in Switzerland, the assessments will take place at one unit only. This ensures quality and standardization, but might result in smaller sample size as participants have to travel to the pediatric unit. To overcome this issue, small incentives will be offered to all participants and travel costs will be reimbursed. Second, study participation includes three assessments at the hospital as well as a supervised first training session and the intervention phase, where participants have to train at home. Therefore, the study design itself could introduce selection bias, indicating that motivated children and adolescents and participants from parents with high engagement are more likely to participate. Although, in contrast, a computerized home-based training can rather be regarded as a low-threshold intervention, it might be that the motivation to participate could have an influence on study results.

## Conclusion

With increasing survival rates of pediatric cancer, evidence-based training for the treatment of cognitive deficits is of major clinical relevance. Currently, the existing empirical evidence on treatment approaches is too limited to form best practices. Therefore, this clinical trial using a combination of neuropsychological and imaging data, will offer new perspectives into the understanding of training-related structural and functional plasticity in the developing brain. Furthermore, we aim to define the neural processes taking place during the course of working memory training and physical exercise and hope to present reliable evidence for a training effect on a neural basis. Such insights into training-related plasticity in the developing brain might help to design tailored rehabilitation programs for pediatric cancer survivors and therefore give valuable insights beyond the two investigated interventions.

## Abbreviations

BT: Brain tumors
CNS: Central nervous system
EF: Executive functions
IQ: Intelligence quotient
